# Combination of Epicatechin 3-Gallate from* Euphorbia hirta* and Cefepime Promotes Potential Synergistic Eradication Action against Resistant Clinical Isolate of* Pseudomonas aeruginosa*

**DOI:** 10.1155/2018/5713703

**Published:** 2018-07-11

**Authors:** Shanmugapriya Perumal, Roziahanim Mahmud, Nornisah Mohamed

**Affiliations:** School of Pharmaceutical Sciences, Universiti Sains Malaysia, 11800 Penang, Malaysia

## Abstract

*Pseudomonas aeruginosa* is naturally resistant to many classes of antipseudomonal antibiotics due to the species ability to easily acquire resistance. Plant-based antibacterial agent in combination with the existing antibiotic proposes an alternative treatment regimen for the eradication of resistant bacterial infections. The antibacterial effects of the isolated epicatechin 3-gallate compound from* Euphorbia hirta* in combination with cefepime were investigated* in vitro* against resistant* P. aeruginosa*. The fractional inhibitory concentration index of the combination was determined using checkerboard broth microdilution method. Epicatechin 3-gallate combined with cefepime had produced synergistic effect against* P. aeruginosa *(with average FIC index of 0.24). The MIC of epicatechin 3-gallate was effectively reduced to MIC/4, MIC/8, MIC/16, and MIC/32 in the presence of cefepime. Time-kill study of epicatechin 3-gallate combined with cefepime exhibited remarkable bactericidal activity where the eradication of* P. aeruginosa *occurred within 4 h of treatment. Scanning electron micrographs revealed apparent cell membrane damage and leakage of cytoplasmic contents from* P. aeruginosa* cells which eventually led to the cell lysis after the combination treatment of epicatechin 3-gallate and cefepime. The potential of epicatechin 3-gallate to act synergistically with cefepime against clinically resistant* P. aeruginosa* strain possibly will maximize the successful outcomes when choosing empirical antibiotic treatment in hospitals or health care institutions.

## 1. Introduction

Since the emergence of multidrug resistant strains (MDR),* Pseudomonas aeruginosa* has gained standing for its competence to cause numerous hospital associated outbreaks. Resistant* P. aeruginosa* pathogen causes severe nosocomial infections especially in immunocompromised and immunocompetent patients [1]. These MDR strains limit the current treatment options of antibiotics with antipseudomonal activity such as ceftazidime, carbapenems, ciprofloxacin, levofloxacin, aztreonam, aminoglycosides, ureidopenicillins, and ticarcillin. A fourth generation cephalosporin antibiotic, cefepime, is used widely as antipseudomonal drug throughout the globe. Although cefepime is frequently employed as a first-line antibiotic against most of the pseudomonal infections, the efficacy of this drug is often challenged over the mass mutation of ever evolving multidrug resistance (MDR) strains [2]. The hyperproduction of the efflux system Mex-XY-OprM and overexpression of Mex-CD-OprJ system in* P. aeruginosa *cause increased resistance to cefepime and other compounds (macrolides, fluoroquinolones) [3, 4]. Several mutations at DNA and protein levels were observed to be the reason behind the hyperproduction of these efflux system regulators [5]. In addition,* P. aeruginosa *naturally acquires resistance to cephalosporins through the constitutive hyperproduction of inducible chromosome-encoded AmpC *β*-lactamase [6].

As the monotherapy often induce failure due to rapid emergence of resistance, dual antibacterial coverage is opt to eradicate* P. aeruginosa *infections. Besides inhibiting the arrival of resistant strains, antibacterial combination regimen is also able to enhance clinical efficacy and broaden the antibacterial activity spectrum in contrast to monotherapy [7]. Additionally, the dosing regimen for the individual antibiotic is lesser in the combination therapy and this may possibly reduce the unwanted side effects of drugs. Up to the present, only scarce articles reported the antibacterial activities of cefepime in combination with other phytocompounds. Earlier, Hemaiswarya [8] had revealed synergism between plant secondary metabolites and clinical antibiotics against few MDR strains. Previous study has described the presence of synergistic activity between* Beilschmiedia cinnamomea* crude extract with cefepime against* P. aeruginosa *(MDR) strain [9].

Combination of antibacterial agents produces synergistic effect when the joint activity of two or more antibacterial agents yields greater effect than their individual activities. In contrast, antagonistic effect exists when the combination of the antibacterial agents provides an effect less than the effect of the individual agents. Indifference effect results when the combination of the antibacterial agents provides an effect equal to the effect of the single antibacterial agent alone. The fractional inhibitory concentration (FIC) index is most frequently employed in laboratory to analyze any* in vitro* synergism, antagonism, or indifference effect between antimicrobial agents. The FIC indices are generally determined using broth microdilution checkerboard method. It represents the sum of FICs of each drug tested. The FIC of each drug is established by dividing the MIC of each drug when used in combination by the MIC of each drug when used alone [10]. Fundamentally, the FIC index is entrenched from Loewe additivity zero-interaction theory [11]. The basis of this theory lays on the hypothesis that a combination of two different drugs will always result in synergy interaction if the FIC index is lower than 1. However, due to the reproducibility errors raised from twofold drug dilution method and the 1-dilution error of single-drug susceptibility testing, the FIC index lower than 0.5 (Σ FIC ≤ 0.5) was restricted to interpretations of synergy [12].

Earlier, we have reported the bioactivity-guided isolation of epicatechin 3-gallate (ECG) from* Euphorbia hirta* (L.) and the mechanism of action of ECG against resistant clinical isolate of* P. aeruginosa* [13, 14]. It is conceivable that combination of secondary metabolites with conventional antibiotics will enhance the antibacterial spectrum to treat resistant strains. Thus, the present study is sought to explore the potential synergistic effect of ECG, previously isolated from* E. hirta* (L.), in combination with cefepime against clinically resistant* P. aeruginosa* isolate.

## 2. Materials and Methods

### 2.1. Bacterial Strain

Resistant clinical isolate of* P. aeruginosa,* strain BF998/11, was of clinical specimen (endo-tracheal tube secretion) collected at USM Hospital, Kubang Kerian in 2013. The specimen was later submitted to the Department of Medical Microbiology and Parasitology (JTMP), School of Medical Sciences, Universiti Sains Malaysia, where it was obtained for this study. The isolate was maintained frozen at -80°C and stored in tryptic soy broth containing 20 % glycerol.

### 2.2. Antibacterial Agent

Laboratory-grade standard powder of cefepime (lot no: 341037; International Laboratory, San Francisco, USA) was obtained and reconstituted according to the manufacturer's instruction. Freshly prepared stock solutions of cefepime were stored under refrigeration until the time of use and discarded after 24 h, as recommended by the manufacturer.* Pseudomonas aeruginosa* (ATCC 27853) was used as a reference strain for the quality control of cefepime (MIC; 2 *µ*g/mL) on the day of the experiment.

### 2.3. Inoculum Preparation

Tryptic soy agar (Difco, USA) was used to recover* P. aeruginosa* culture. Well isolated colony of* P. aeruginosa* was transferred into sterile cation-adjusted Mueller Hinton broth (CAMHB) (HiMedia, India) to obtain a liquid suspension (5 mL) that matches McFarland 0.5 turbidity standards. This standardized inoculum was immediately diluted at 1:100 ratio in CAMHB to yield 1 x 10^6^ CFU/mL. The subsequent 1:2 dilution with test compounds in the checkerboard assay will bring the final inoculum or test concentration of bacteria to 5 x 10^5^ CFU/mL.

### 2.4. Checkerboard Broth Microdilution

The MICs of ECG and cefepime were reported previously using broth microdilution assay [13]. To determine the fractional inhibitory concentration (FIC) between ECG and cefepime, the checkerboard broth microdilution method has been employed. This assay was performed using flat-bottomed polystyrene 96-well clear microtitre plates (Greiner Bio-One, Germany). The combination of ECG and cefepime was evaluated against clinically resistant* P. aeruginosa* strain. The concentration of stock solutions of ECG as well as cefepime was prepared five times superior to the highest concentration to be tested. Seven doubling dilutions of cefepime (2.0-128.0 *µ*g/mL) and seven doubling dilutions of ECG (2.0-128.0 *µ*g/mL) were tested. To construct the ECG-cefepime combination, seven twofold dilutions of ECG were made with CAMHB in the grid of seven rows by seven columns. Firstly, about 50 *µ*L of sterile CAMHB was dispensed into every well (64 wells). An amount of 50 *µ*L of ECG solution was added individually in row H8-H2 at descending concentration (128.0-2.0 *µ*g/mL). A starting concentration of 256 *µ*g/mL of ECG (50 *µ*L) was added to wells in the column A8-G8. Serial dilution was made horizontally along the* x*-axis from column 8 to column 2 which results in decreasing concentration of ECG (128.0-2.0 *µ*g/mL). The excess medium (50 *µ*L) from column 2 was discarded.

An amount of 50 *µ*L of cefepime solution was added individually in column A1-G1 at descending concentration (128.0-2.0 *µ*g/mL). Twofold dilutions of cefepime (128.0-2.0 *µ*g/mL) were then added to row A-G of each column (2-8) containing ECG solution. Row H contained no cefepime. Thus, each of the 64 wells employed for the assay possessed an exclusive combination of concentrations of two antibacterial agents. Well which contained only media and inoculum (no antibacterial agent) was used as a positive growth control (H1) and three wells were used as sterility control (negative control) which only contained media (CAMHB) and without inoculum. After the antibacterial agent dilution, each well in the microplates was inoculated with 100 *µ*L of* P. aeruginosa* suspension forming a final concentration of 5x10^5^ CFU/mL. The broth microdilution checkerboard plate illustrating the final testing concentration for the combination of ECG and cefepime after the bacterial inoculation is shown in Supplementary Figure S1. The microplates were then incubated overnight (24 h) in ambient air at 37°C. After the 24 h incubation, the checkerboard microdilution plate was added with 50 *μ*L of INT (0.2 mg/mL) in all the wells and incubated for a further 1 h at 37°C. The MIC of the antibacterial agents was examined visually by observing a colour change of INT from yellow to red indicating bacterial growth. No colour changes signify nil bacterial growth due to antibiotic inhibition. The MIC of individual agent, ECG, was read along row H (the lowest concentration that inhibits the growth of bacteria); meanwhile, MIC of cefepime was read along the column 1. The checkerboard assay was performed three times independently on different days, each time in duplicate. To determine the FIC index, the wells of the microplates that corresponds to an MIC (growth inhibition) was observed. The sum of ΣFIC was calculated for each well with the following equation:

(1) ΣFIC=FICA+FICB=MICAB/MICA+MICBA/MICB

MIC_AB_ was described as the MIC of drug A in the presence of drug B, where the concentration of the drug A in the well is the lowest inhibitory concentration in its row. MIC_BA_ was described as the MIC of drug B in the presence of drug A, where the concentration of the drug B in the well is the lowest inhibitory concentration in its column. The FIC index for* P. aeruginosa* was determined by summing up ΣFICs of each well that showed growth to no growth interface on the checkerboard plate divided by the total number of the wells (n) for which FICs were ascertained as per (2):

(2) $$\begin{array}{c} \left(\;\Sigma {FIC}_{1}+\Sigma {FIC}_{2}+\ldots \frac{\Sigma {FIC}_{n}}{n}\;\right) \end{array}$$

The interaction was interpreted as synergy if average FIC index is ≤ 0.5 [12].

### 2.5. Time-Kill Curve

The time-kill method was performed in accordance with guidelines provided by the Clinical and Laboratory Standards Institute [15]. The time-kill curve determined the rate of killing of* P. aeruginosa* within 24 h of treatment by ECG and cefepime, used alone (1 x MIC) and in combination (ECG; 2*µ*g/mL and cefepime; 1 *µ*g/mL). Briefly, the antibacterial agent(s) were suspended in CAMHB followed by inoculation of* P. aeruginosa* suspension at 5 x 10^5^ CFU/mL. The treated culture medium was incubated at 37°C. An aliquot of 1 mL was collected at selected time intervals of 0.5, 4, 8, 12, and 24 h of incubation period and serially diluted (10-fold) in normal saline. An amount of 100 *µ*L from each dilution was inoculated on Mueller Hinton agar and the plates were incubated at 37°C for 24 h. Colonies formed on the agar plates were enumerated and CFU/mL was determined. The lower limit of detection was 300 CFU/mL. The results presented are the mean of triplicate determinations.

### 2.6. Scanning Electron Microscopy

The influence of ECG and cefepime treated alone and in combination on the cell morphology of* P. aeruginosa* was investigated by scanning electron microscopy (SEM). An amount of 100 *µ*L of cell suspension of* P. aeruginosa *(5 x 10^7^ CFU/mL) was inoculated into 10 mL of CAMHB containing ECG and cefepime, present individually at 1×MIC and in combination (ECG; 2*µ*g/mL and cefepime; 1 *µ*g/mL). These treated tubes were incubated at 37°C for 4 h, 8 h, and 12 h, respectively. Control sample which did receive treatment was as well run in parallel. The harvested bacterial pellets obtained after the treatment were fixed with McDowell-Trump fixative in 0.1 M phosphate buffer (pH 7.2) for 24 h at 4°C. The fixed cells were washed twice in 0.1 M phosphate buffer (pH 7.2). Postfixation was carried out in 1% buffered osmium tetroxide (OSO_4_) for 1 h, washed with distilled water twice, and performed the dehydration process in graded solutions of ethanol series (50%, 75%, 95%, and 100%) for 10 min in each alcohol and finally with hexamethyldisilazane (HMDS) for 10 min. The dried cells were mounted on to a SEM stub and coated with gold sputtering (60 s, 1.8 mA, 2.4 kV) in a Polaron SEM, SC515 coating system. The samples were examined in a LEO SUPRA 50VP SEM (Carl Zeiss, FESEM, Oberkochen, Germany) to assess the changes in cell morphology. Images were captured at low and high magnifying cation to accommodate details of 3D-cell morphology.

## 3. Results

### 3.1. FIC Index of ECG and Cefepime Combination

**Figure 1:**
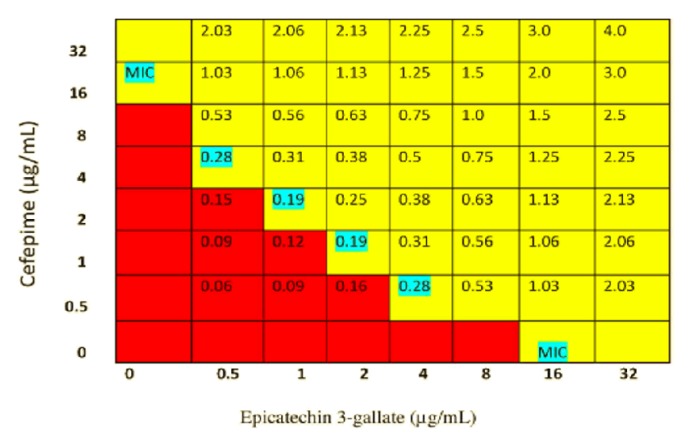
Schematic representation of the* in vitro* synergistic interaction in epicatechin 3-gallate and cefepime combination against* Pseudomonas aeruginosa*. Numbers in the checkerboard represent FIC index (ΣFIC) for each combined concentration of antibacterial agents. The highlighted numbers are FIC indices (synergistic range) for the lowest concentrations of the antibacterial combination in each row and column where growth of* P. aeruginosa* is inhibited. The average ΣFIC for the cefepime and ECG combination is 0.24.

Different concentration combinations of ECG and cefepime were tested to enhance the growth inhibition activity of clinically resistant* P. aeruginosa* isolate. The efficiency of this combination was evaluated using checkerboard broth microdilution method. The previous study reported MIC values of ECG and cefepime used alone were 16 *µ*g/mL each [13]. In the present study,* in vitro* combination of ECG and cefepime had produced synergistic effect. The MIC for ECG was reduced to MIC/4, MIC/8, MIC/16, and MIC/32 in the presence of cefepime at the concentration of 0.5, 1, 2, and 4 *µ*g/mL, respectively. The first well of the lowest concentrations in each row and column where no growth can be seen on the checkerboard plate was utilized to determine the average FIC index for* P. aeruginosa*. The FIC index analysis of ECG and cefepime combination resulted in ΣFIC ranging from 0.19 to 0.28. This combination was synergistic against* P. aeruginosa *with average FIC index of 0.24. To visualize the synergistic ranges and the wells engaged in this range, a schematic representation of the checkerboard plate containing ECG and cefepime combination is shown in Figure 1 together with the two MICs, the ΣFICs for each well and the synergistic range (highlighted ΣFICs).

### 3.2. Time-Kill Analysis

**Figure 2:**
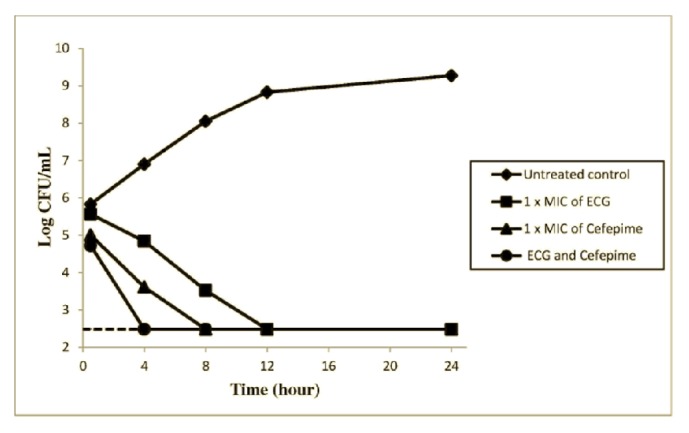
Time-kill curves of epicatechin 3-gallate (ECG) and cefepime, used alone and in combination against resistant clinical isolate of* Pseudomonas aeruginosa*. Each point represents the means triplicate determinations. The limit of detection (300 CFU/mL) is indicated by the dashed lines.

The* in vitro* time-kill analyses of ECG and cefepime used alone and in combination are shown in Figure 2. The growth of* P. aeruginosa *was fully inhibited by ECG at the concentration of 1 x MIC (15.6 *µ*g/mL) in 12 h of incubation period. The initial bactericidal effect took place at exposure time of 0.5 h, where viable counts decline from log 5.7 to log 5.56 within 30 min and continuously decline until 24 h. In contrast, cefepime at the same concentration eradicates* P. aeruginosa *growth within 8 h of treatment. The combination of ECG and cefepime showed remarkable bactericidal activity where the killing of* P. aeruginosa *occurred within 4 h of treatment.

### 3.3. Cell Morphology of Treated* P. aeruginosa* by SEM

**Figure 3:**
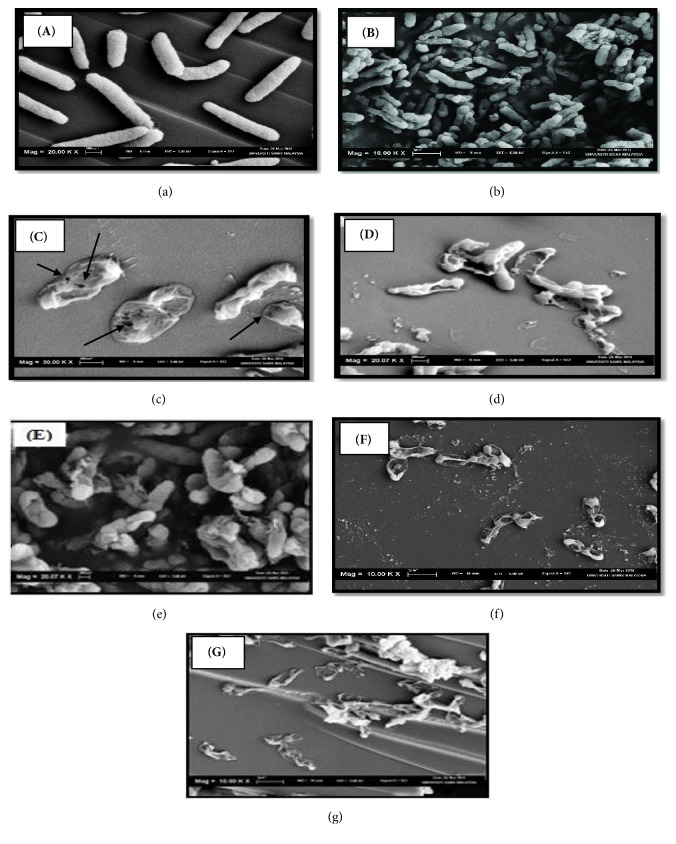
Scanning electron micrographs of* Pseudomonas aeruginosa*. (a) Untreated control cells seem to be healthily proliferating and appeared as rods with smooth cell surface which indicates intact cell wall and well-defined plasma membrane. ((b), (c), (d)) Treated* P. aeruginosa* cells by epicatechin 3-gallate compound for 4, 8, and 12 h. (b) Surface structure of* P. aeruginosa* cells appeared corrugated after 4 h of treatment, (c) punctures and dents observed on the cell surface signify membrane integrity disruption over 8 h of treatment, and (d) excessive leakage of cellular contents ultimately led to total cell rupture after 12 h of treatment. Arrows indicate membrane rupture (holes) on the cells. ((e), (f)) Cells treated by cefepime for 4 and 12 h. (e) Extreme cell shrinkage with multiple punctures observed at 4 h of cefepime treatment. (f) Complete cell death achieved upon 8 h of cefepime exposure. (g)* P. aeruginosa* cells treated with epicatechin 3-gallate and cefepime combination at 4 h. Absolute cell lysis attained within 4 h of combination regimen.

The SEM images of untreated cells (control) appeared to be rod-shaped and with smooth cell surface (Figure 3(a)). Control cells seemed healthily proliferating with intact cell walls and well-defined plasma membranes. There was no noticeable disruption on the rigidity of cell wall and plasma membranes before* P. aeruginosa* cells were treated. Following 4 h of treatment with ECG,* P. aeruginosa* cell surface structure appeared corrugated and the bacteria appeared shorter which indicate the growth inhibition (Figure 3(b)). With subsequent treatment with the ECG for 8 h,* P. aeruginosa* cells could not maintain the integrity of outer membrane and plasma membrane. This is evident in Figure 3(c) as there were decreased cells surface stiffness and slight leakages of cellular cytoplasmic substances. The latter treatment of* P. aeruginosa* cells with ECG for 12 h had induced cell rupture and eventually released all the cellular contents (Figure 3(d)). Cefepime treated cells attained death upon 8 h of treatment (Figure 3(f)). There were multiple punctures observed on cefepime treated* P. aeruginosa* cells at 4 h of treatment (Figure 3(e)). Meanwhile, combination of ECG and cefepime regimen had expressed the* P. aeruginosa* cell lysis within 4 h (Figure 3(g)).

## 4. Discussion

Antibacterial combinations are being researched extensively nowadays to discover the synergistic combo interaction among potential agents. In the present study, cefepime combined with ECG isolated from* E. hirta* plant had produced synergistic eradication effect against resistant clinical isolate of* P. aeruginosa*. Associating cefepime with ECG allowed lower concentrations of cefepime and ECG to be used than when each substance molecule was taken alone. The MIC for ECG was reduced to 4, 2, 1, and 0.5 *µ*g/mL in the presence of cefepime at the concentration of 0.5, 1, 2, and 4 *µ*g/mL, respectively. The current finding had confirmed that synergism among drugs used would enhance the activity of the individual drug involved in the combination. Specific combination of antibacterial agents is known to have synergistic interactions if the effect of the combination is greater than the effect of either the agent alone or the sum of the effects of the individual agents [16]. This is evident in the work of Hu [17] that had explained the superior activity of epigallocatechin gallate (EGCG) against methicillin-resistant* S. aureus* (MRSA) when combined with ampicillin. This study contrasts with the work reported by Stapleton [18] in which ECG, EGCG, and CG extracted from Japanese green tea (*Camellia sinensis*) had the capacity to reverse oxacillin resistance against MRSA when used individually. However, the inhibitory activity was potentially stronger in the presence of combination therapy.

The potential synergistic interactions between herbs and prescribed drugs could be higher than drug-drug combination because drugs usually contain single chemical entities, while almost all herb products contain mixtures of pharmacologically active constituents [19]. Traditionally in South Africa, medicinal plants are used in combination to optimize their efficacy. For example,* Eriocephalus africanus *(Cape snowbush) is often blended with* Santalum album* (sandalwood) or* Citrus bergamia* (bergamot) and the resulting mixtures are used for the treatment of colds, flu, depression, muscular aches, and pain [20]. The combination of* Agathosma betulina* (buchu) with* Lavandula angustifolia* (lavender) is habitually applied to wounds infections and burn in India [21].

Nevertheless, the synergistic combination of various antibacterial agents is often needed in the treatment of serious infections. The characteristics and the coverage of the drugs interaction are typically established in* in vitro* studies. The checkerboard technique is a common laboratory approach used to verify synergism, antagonism, and indifference by means of fractional inhibitory concentrations (FIC) indices [22]. In this method, two antibacterial agents are tested in serial dilutions to find the concentration of each antibiotic, both alone and in combination that produces specified interactions. The microdilution checkerboard method employed in this study concerns the variability engaged in the inoculum size along with involvement of inappropriate drug concentrations [23]. Therefore, to test reproducibility, the same checkerboard experiment for the antibacterial agents' combination was conducted and repeated on three distinctive days. No discrepancy was found, and the result proved reproducible.

In the time-kill study, the combination of ECG and cefepime demonstrated remarkable bactericidal activity where the killing of* P. aeruginosa *occurred within 4 h of treatment in contrast to the treatment of ECG and cefepime alone. Previously, Rizvi [24] had mentioned that to optimize the chance of clinical success and to reduce the cases of emergence of resistance, an adequate initial combination therapy of antibacterial agents should be administered in a timely manner. Earlier, Perumal [14] had reported the mechanism of action of ECG against* P. aeruginosa*. The findings of the study disclosed ECG isolated from* E. hirta* plant acted as an antibacterial compound by targeting both cell wall and cytoplasmic membrane of* P. aeruginosa. *The increased membrane permeability of* P. aeruginosa* by ECG had facilitated the access of hydrophobic antibiotics, release of potassium ions, and leakages of nucleotides, thus leading to cell lysis [14].

The SEM micrographs showing ECG alone caused apparent cell membrane damage and leakages of cytoplasmic contents from* P. aeruginosa* which eventually led to the cell death after 12 h of treatment, whereas the combination regimen of ECG and cefepime had produced significant synergistic effect in the rapid killing of* P. aeruginosa *within 4 h of exposure. The synergistic effect observed between cefepime and ECG can be due to the different target site or mode of action of each respective antibacterial agent involved [25]. The cell morphology study by SEM showed that ECG had increased the permeability of the cell wall where the leakage of cytoplasmic content was apparent; meanwhile, cefepime being *β*-lactam antibiotic actively inhibited the synthesis of cell wall material of* P. aeruginosa *[26].

## 5. Conclusions

In conclusion, the* in vitro* combination of cefepime and ECG resulted in synergism that yielded remarkable antibacterial activity. The findings propose the likelihood of herb-drug combination for reducing the dose of antibiotics needed to treat* P. aeruginosa* infection. The potential of cefepime to act synergistically with ECG against clinically resistant* P. aeruginosa* strain might exhibit beneficial when choosing empirical antibiotic treatment in hospitals or health care institutions. Moreover, the combination of cefepime with ECG suggests an attractive prospect for the development of new chemotherapeutic strategies for pseudomonal infections. Prerequisite animal and clinical studies are further required to ascertain this combinatorial therapy (ECG and cefepime) in selected clinical conditions for instance pneumonia, dermatitis, ulcerative keratitis, otitis externa, gastrointestinal infections, bloodstream infections, urinary tract infections, and respiratory system infections.

## Data Availability

The data used to support the findings of this study are available from the corresponding author upon request.

## Supplementary Materials

Supplementary Materials Figure S1: broth microdilution checkerboard plate containing respective combination of concentrations (*μ*g/mL) of epicatechin 3-gallate and cefepime. E: epicatechin 3-gallate and Cef: cefepime. The final concentrations of epicatechin 3-gallate and cefepime were 0.5, 1, 2, 4, 8, 16, and 32 *μ*g/mL, respectively.
