# Supplementary material for: Combination of Epicatechin 3-Gallate from Euphorbia hirta and Cefepime Promotes Potential Synergistic Eradication Action against Resistant Clinical Isolate of Pseudomonas aeruginosa
Source: Evid Based Complement Alternat Med. 2018 Jul 11;2018:5713703. doi: 10.1155/2018/5713703 (PMC6077534; doi:10.1155/2018/5713703)
Supplement: Supplementary Materials — Figure S1: broth microdilution checkerboard plate containing respective combination of concentrations (μg/mL) of epicatechin 3-gallate and cefepime. E: epicatechin 3-gallate and Cef: cefepime. The final concentrations of epicatechin 3-gallate and cefepime were 0.5, 1, 2, 4, 8, 16, and 32 μg/mL, respectively. [file 5713703.f1.pdf]

	1	2	3	4	5	6	7	8
**A**	Cef 32	E 0.5 + Cef 32	E 1 + Cef 32	E 2 + Cef 32	E 4 + Cef 32	E 8 + Cef 32	E 16 + Cef 32	E 32 + Cef 32
**B**	Cef 16	E 0.5 + Cef 16	E 1 + Cef 16	E 2 + Cef 16	E 4 + Cef 16	E 8 + Cef 16	E 16 + Cef 16	E 32 + Cef 16
**C**	Cef 8	E 0.5 + Cef 8	E 1 + Cef 8	E 2 + Cef 8	E 4 + Cef 8	E 8 + Cef 8	E 16 + Cef 8	E 32 + Cef 8
**D**	Cef 4	E 0.5 + Cef 4	E 1 + Cef 4	E 2 + Cef 4	E 4 + Cef 4	E 8 + Cef 4	E 16 + Cef 4	E 32 + Cef 4
**E**	Cef 2	E 0.5 + Cef 2	E 1 + Cef 2	E 2 + Cef 2	E 4 + Cef 2	E 8 + Cef 2	E 16 + Cef 2	E 32 + Cef 2
**F**	Cef 1	E 0.5 + Cef 1	E 1 + Cef 1	E 2 + Cef 1	E 4 + Cef 1	E 8 + Cef 1	E 16 + Cef 1	E 32 + Cef 1
**G**	Cef 0.5	E 0.5 + Cef 0.5	E 1 + Cef 0.5	E 2 + Cef 0.5	E 4 + Cef 0.5	E 8 + Cef 0.5	E 16 + Cef 0.5	E 32 + Cef 0.5
**H**	0	E 0.5	E 1	E 2	E 4	E 8	E 16	E 32

Figure S1: Broth microdilution checkerboard plate containing respective combination of concentrations (μg/mL) of epicatechin 3-gallate and cefepime. E; epicatechin 3-gallate and Cef; cefepime. The final concentration of epicatechin 3-gallate and cefepime were 0.5, 1, 2, 4, 8, 16 and 32 μg/mL respectively.
